# Mouse SPNS2 Functions as a Sphingosine-1-Phosphate Transporter in Vascular Endothelial Cells

**DOI:** 10.1371/journal.pone.0038941

**Published:** 2012-06-12

**Authors:** Yu Hisano, Naoki Kobayashi, Akihito Yamaguchi, Tsuyoshi Nishi

**Affiliations:** 1 Department of Cell Membrane Biology, Institute of Scientific and Industrial Research, Osaka University, Ibaraki, Osaka, Japan; 2 Graduate School of Pharmaceutical Sciences, Osaka University, Suita, Osaka, Japan; Cornell University, United States of America

## Abstract

Sphingosine-1-phosphate (S1P), a sphingolipid metabolite that is produced inside the cells, regulates a variety of physiological and pathological responses via S1P receptors (S1P1–5). Signal transduction between cells consists of three steps; the synthesis of signaling molecules, their export to the extracellular space and their recognition by receptors. An S1P concentration gradient is essential for the migration of various cell types that express S1P receptors, such as lymphocytes, pre-osteoclasts, cancer cells and endothelial cells. To maintain this concentration gradient, plasma S1P concentration must be at a higher level. However, little is known about the molecular mechanism by which S1P is supplied to extracellular environments such as blood plasma. Here, we show that SPNS2 functions as an S1P transporter in vascular endothelial cells but not in erythrocytes and platelets. Moreover, the plasma S1P concentration of SPNS2-deficient mice was reduced to approximately 60% of wild-type, and SPNS2-deficient mice were lymphopenic. Our results demonstrate that SPNS2 is the first physiological S1P transporter in mammals and is a key determinant of lymphocyte egress from the thymus.

## Introduction

Sphingosine-1-phosphate (S1P), a bioactive sphingolipid that is recognized by five G protein-coupled receptors (S1P1–5) and plays a key role in angiogenesis, bone homeostasis and the immune system [1], [2], [3], [4], [5], [6]. Because S1P receptors are located on the cell surface, S1P, which is produced in the cell from sphingosine by sphingosine kinases (SPHK1 and SPHK2) and contains a negatively charged phosphate group, must be exported from the cells in a carrier-mediated manner. The production of S1P and its recognition by S1P receptors have been investigated extensively [7], [8], [9], [10]. However, information on S1P secretion from the cells is insufficient.

SPHK1 and SPHK2 produce S1P by the phosphorylation of sphingosine, and S1P is dephosphorylated to regenerate sphingosine by S1P phosphatases (SPPs) and/or extracellular lipid phosphate phosphatases (LPPs) [10], [11], [12], [13]. S1P is also degraded by S1P lyase (SPL), leading to the formation of ethanolamine phosphate and hexadecenal [8], [9]. The amount of S1P is determined by the balance of the activities of the S1P metabolizing enzymes. The S1P concentration in tissue is maintained at lower levels due to S1P-degrading activities [14], [15]. In contrast, S1P in plasma exists mainly at higher concentrations (∼µM), in association with high-density lipoprotein and albumin [16], [17], [18]. An exogenous C_17_-S1P, an S1P analog, is rapidly degraded in plasma (with a half-life of approximately 15 minutes), which indicates that there is an active degradation pathway in plasma; therefore, the high S1P level in plasma must be maintained by a continuous S1P supply from S1P-producing cells [19]. Erythrocytes and platelets have the ability to produce and release S1P into plasma, and erythrocytes play an important, but not exclusive, role in maintaining the plasma S1P levels [19], [20], [21], [22], [23]. Additionally, non-hematopoietic sources of plasma S1P, such as vascular endothelial cells (ECs) or other types of cells, have been proposed [19], [21], [24].

In addition to platelets, erythrocytes and ECs, several other types of cells with the ability to secrete S1P have been identified [22], [23], [24], [25], [26], [27]; however the transporter molecules releasing S1P from the cells into plasma have not yet been identified. Recently, we described that zebrafish Spns2 (zSpns2) in the yolk syncytial layer (YSL) functions as an S1P transporter based on an analysis of the zebrafish mutant *ko157* [28]; human and mouse genomes contain its orthologs. Because YSL is a fish-specific extra-embryonic tissue, however, the physiological role of SPNS2 in mammals has been unclear. In this report, we report that SPNS2 functions as an S1P transporter in ECs and is an essential regulator of lymphocyte egress from the thymus.

## Results

### Disruption of mouse SPNS2, an S1P transporter

**Figure 1:**
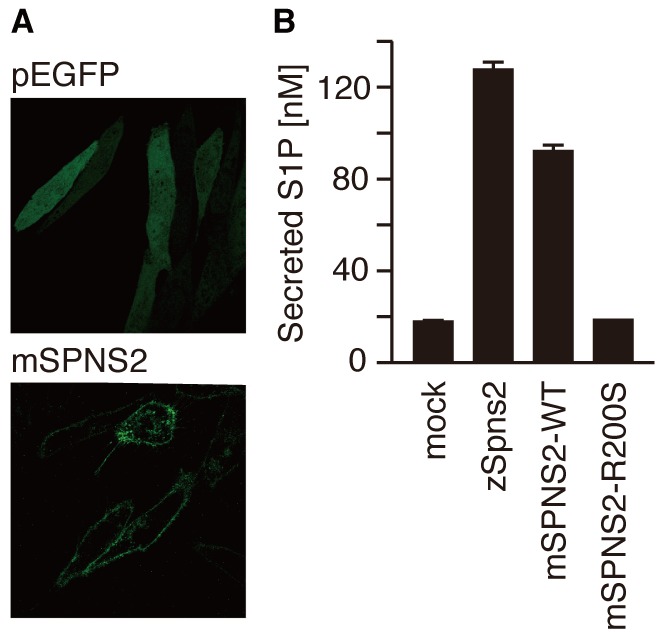
Mouse SPNS2 exports S1P from the cells. (A) Cellular localization of mouse SPNS2. CHO-SPHK1 cells expressing EGFP or mSPNS2-EGFP were observed by confocal fluorescence microscopy (LSM5 Pascal, Carl Zeiss). (B) The endogenous S1P released from CHO-SPHK1 cells expressing EGFP, mSPNS2-EGFP or mSPNS2-R200S-EGFP was separated and quantitated with C_17_-S1P (internal standard) by HPLC. The release of endogenous S1P was observed in mSPNS2-EGFP-transfected cells but not in EGFP-expressing or mSPNS2 (R200S)-EGFP-expressing cells. The graph shows the average values from three experiments, with error bars representing the standard error.

Human and mouse SPNS2 (hSPNS2 and mSPNS2) have high sequence identities with zSpns2 (zSpns2 vs. hSPNS2 or mSPNS2 is 72%; hSPNS2 vs. mSPNS2 is 95%). When hSPNS2 or mSPNS2 were expressed in Chinese hamster ovary (CHO) cells expressing sphingosine kinase (SPHK) 1, the protein localized to the plasma membrane and exported S1P in a manner similar to zSpns2 (Figure 1) [28], [29]. We analyzed SPNS2-deficient mice to examine the physiological role of mammalian SPNS2.

**Figure 2:**
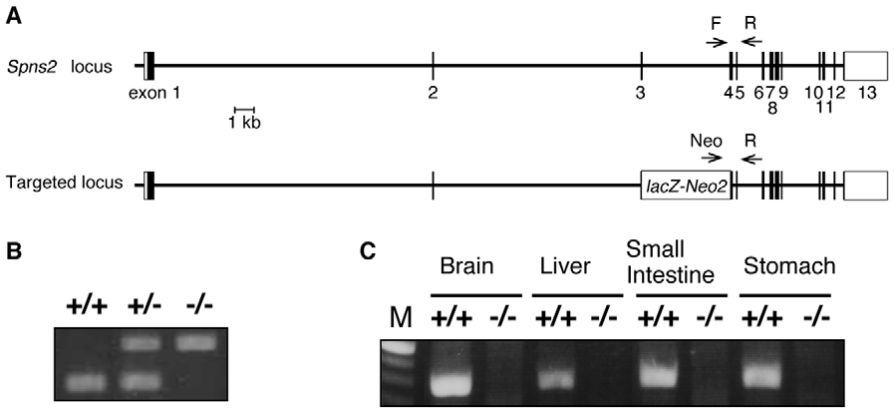
SPNS2-deficient mice. (A) Targeting scheme to generate the *Spns2* genomic deletion allele. The exons of the putative coding and noncoding regions are shown as black and white boxes, respectively. The *LacZ-neo^r^* cassette in the deleted allele is indicated with a white rectangle. The primers used for genotyping are indicated by arrows. (B) Deletion of mouse *Spns2* from the genome was confirmed with PCR using genomic DNA isolated from *Spns2* ^+/+^, *Spns2* ^+/−^ and *Spns2* ^−/−^ mouse tails. (C) Knock-out of mouse *Spns2* was confirmed by conventional RT-PCR using mRNA isolated from *Spns2* ^+/+^ or *Spns2^−/−^* mouse tissues.

SPNS2-deficient mice were generated by the disruption of exon 3–4, which contains the codon encoding Arg200, an amino acid that is essential for S1P export activity (Figure 1 and Figure S1) [28]. *Spns2* deficiency was confirmed by the absence of *Spns2* exons and mRNA (Figure 2). Although SPNS2-deficient mice were born in the expected Mendelian ratios, they displayed an eye-open at birth (EOB) phenotype, and approximately 40% of them succumbed to cryptogenic death at 4 to 5 weeks of age (Figure S2). Therefore, we used 4-week-old mice for our studies to avoid analyzing a biased population of SPNS2-deficient mice. Other than the EOB phenotype, the SPNS2-deficient mice showed no abnormalities in the cardiovascular system or other organs, suggesting that there are functional differences between zebrafish and mammals in the physiological roles of SPNS2 in cardiogenesis.

### SPNS2-deficient mice showed a decrease in S1P plasma levels

**Figure 3:**
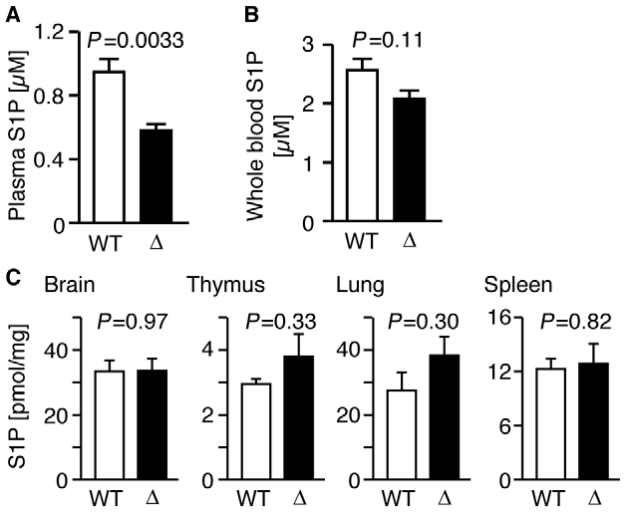
Plasma S1P concentration is decreased in SPNS2-deficient mice. (A) Concentration of plasma S1P in wild-type (WT, n = 14) and SPNS2-deficient mice (Δ, n = 9). The *P*-value from comparisons between WT and Δ samples is indicated. (B) Concentration of whole blood S1P in wild-type (WT, n = 7) and SPNS2-deficient mice (Δ, n = 5). (C) S1P contents of mouse tissues. S1P contents of brain, thymus, lung and spleen from wild-type (WT, n = 3) or SPNS2-deficient (Δ, n = 3) mice were measured by HPLC. C_17_-S1P was used as the internal standard. Graphs show the average values from multiple experiments, with error bars representing the standard error. The *P*-values of comparisons between WT and Δ samples is indicated.

The S1P concentration in the plasma of SPNS2-deficient mice was approximately 60% of that observed in wild-type mice, while the S1P concentration in the whole blood fraction (including blood cells) showed no significant difference (Figure 3A and 3B), suggesting that SPNS2 plays a significant role in maintaining the S1P level in plasma by exporting S1P from S1P-producing cells into the plasma. In various organs (thymus, spleen, lung and brain), the S1P level showed no significant differences between wild-type and SPNS2-deficient mice (Figure 3C). Because the S1P concentration in these organs reflects the amount of intracellular S1P, we concluded that SPNS2 does not affect the production or degradation of intracellular S1P.

### SPNS2-deficient mice showed a deficiency in thymocyte egress

**Figure 4:**
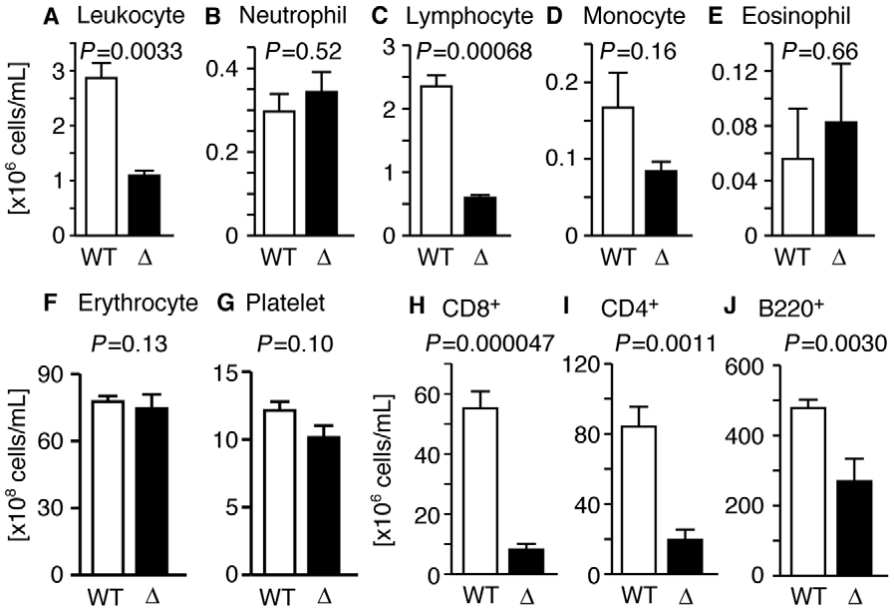
SPNS2 is required for normal lymphocyte egress. (A–G) Blood from wild-type (WT, n = 3) and SPNS2-deficient mice (Δ, n = 3) was collected, and leukocytes, leukocyte subpopulations, erythrocytes and platelets were counted using flow cytometry. Bar graphs show the average values from three experiments. (H–J) Flow cytometric analysis of blood from wild-type (WT, red, n = 13) and SPNS2-deficient (Δ, blue, n = 7) mice. CD8 (H), CD4 (I) and B220 (J) were used to detect each cell type. Graphs show the average values from multiple experiments, with error bars representing the standard error. The *P*-value of comparisons between WT and Δ samples is indicated.

One of the most remarkable physiological roles of S1P receptors is the regulation of lymphocyte egress from lymphoid organs into the blood [30]. Thus, we examined whether SPNS2 supplies S1P which is recognized by lymphocyte S1P1 and regulates their egress. The blood of SPNS2-deficient mice contained significantly fewer leukocytes (Figure 4A and Table S1), while the numbers of erythrocytes and platelets were not changed (Figure 4F and 4G, and Table S1). Within the leukocyte subpopulation, the number of lymphocytes was drastically decreased, while the numbers of neutrophils and eosinophils were unchanged (Figure 4B–4E). The number of monocytes in SPNS2-deficient mice was decreased, but did not show statistical significance (Figure 4C). Furthermore, the numbers of circulating CD4^+^ and CD8^+^ T cells were remarkably reduced, and the number of circulating B220^+^ B cells was decreased by half compared with wild-type mice (Figure 4H–4J). These results raised the possibility that the S1P secreted by SPNS2 is essential for lymphocyte circulation. However, there are other possibilities; for example, the maturation of lymphocytes and/or the migration of lymphocytes in response to S1P might be defective in SPNS2-deficient mice.

**Figure 5:**
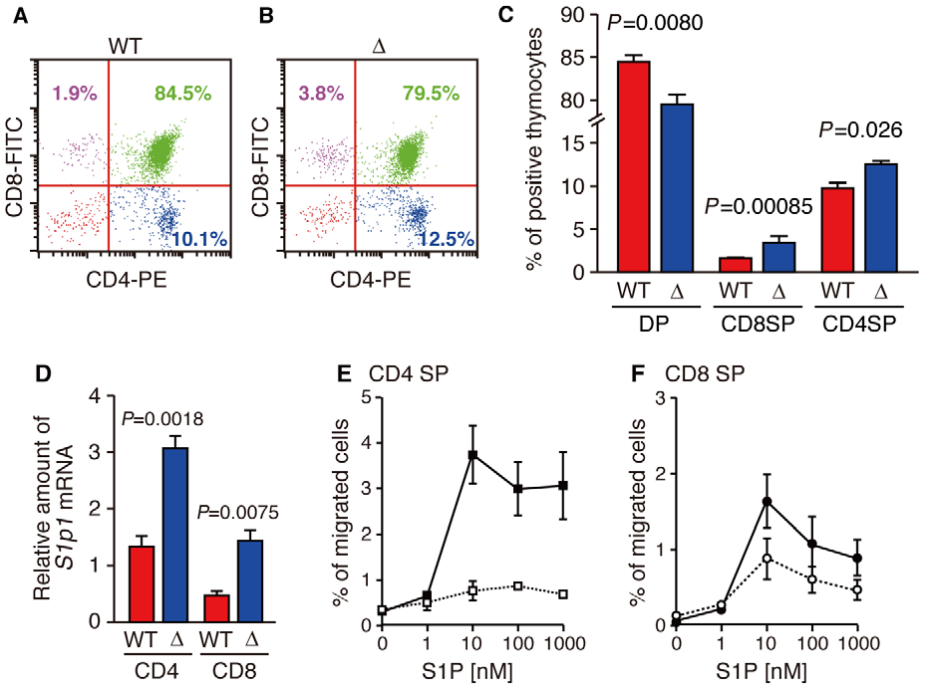
Thymocytes of SPNS2-deficient mice can mature and migrate toward S1P. (A and B) Expression profiles for CD4 and CD8. Thymus-derived CD4^+^ and CD8^+^ cells from wild-type (A, WT, n = 5) and SPNS2-deficient (B, Δ, n = 7) mice were analyzed by flow cytometry. Each plot is representative of multiple experiments. Numbers show the percent of total lymphocytes, identified by their size. (C) The percentage corresponding to CD4^+^ CD8^+^ (DP), CD4^−^ CD8^+^ (CD8SP) or CD4^+^ CD8^−^ (CD4SP) populations. (D) Quantitative analysis of *S1p1* mRNA in mature thymocytes. CD4 or CD8 single positive cells were purified from the thymus of wild-type (WT, n = 3) or SPNS2-deficient (Δ, n = 5) mice with MACS. The amount of *S1p1* mRNA is normalized to that of *Hprt*. The primers and probes used for PCR are indicated in Table S2. The *P*-values from comparisons between WT and Δ samples are indicated. (E, F) Chemotaxis assays of mature thymocytes of wild-type (WT, n = 10) or SPNS2-deficient (Δ, n = 5) mice. The percentage of input cells of the CD62L^hi^ and CD4 (E) or CD8 (F) single positive phenotype that migrated toward S1P is shown. open square, wild-type CD4 single positive; closed square, SPNS2-deficient CD4 single positive; open circle, wild-type CD8 single positive; closed circle, SPNS2-deficient CD8 single positive. The graphs show the average values from multiple experiments, with error bars representing the standard error.

As shown in Figure 5, T cells examined in the thymus of SPNS2-deficient mice, and the population of mature T cells (CD4 or CD8 positive; single positive) in the thymus was increased while that of immature T cells (CD4 and CD8 positive; double positive) was decreased. To examine the ability of mature thymocytes to migrate in response to S1P, the amount of *S1p1* mRNA in mature thymocytes was quantified and their migration activity was measured in a transwell assay. The amount of *S1p1* mRNA in CD4 single positive cells was two to three times higher level than in CD8 single positive cells, and mature thymocytes of SPNS2-deficient mice have more *S1p1* mRNA than wild-type mice, in both CD4 and CD8 single positive cells (Figure 5D). Moreover, CD4 and CD8 single positive cells from SPNS2-deficient mice showed a high migration activity at a lower S1P dose (1 to 10 nM) compared to that of wild-type (10 to 100 nM), presumably due to the increased expression of S1P1 (Figure 5E and 5F). It is possible that the higher amount of *S1p1* mRNA and the increased S1P sensitivity in single positive cells from SPNS2-deficient mice might be caused by compensation for the depletion of S1P required for thymocyte egress. These results indicate that thymocytes of SPNS2-deficient mice can mature and migrate toward S1P normally, but they could not emigrate from the thymus into blood in the absence of SPNS2. Consequently, the number of circulating T cells in the peripheral blood was dramatically reduced in SPNS2-deficient mice.

### SPNS2 does not function in erythrocytes and platelets

**Figure 6:**
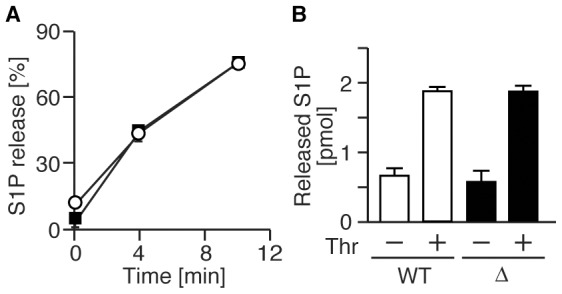
SPNS2 does not function in erythrocytes and platelets. (A) Time-dependent S1P release from erythrocytes. Erythrocytes from wild-type (closed squares, n = 3) and SPNS2-deficient mice (open circles, n = 4) were incubated with [^3^H]sphingosine at 37 °C, and [^3^H]S1P exported into the medium was measured at the indicated times. S1P release is shown as a percentage: (amount of supernatant)/(total amount). (B) Thrombin induced S1P release from platelets. Platelets from wild-type (WT, n = 4) and SPNS2-deficient mice (Δ, n = 4) were incubated in the presence or absence of thrombin, and S1P released into the medium was measured by UPLC-MS/MS. The graphs show the average values from multiple experiments, with error bars representing the standard error.

The release of S1P from platelets and erythrocytes has been compared in detail by ourselves and by other researchers [22], [23], [31], [32], [33], [34]. Erythrocytes and platelets are able to produce and secrete S1P. Erythrocytes predominate among blood cells, and appear to be the major contributor to plasma S1P [35]. Therefore, we measured S1P release activity in erythrocytes isolated from wild-type or SPNS2-deficient mice. There were no differences in S1P release activity (Figure 6A). Platelets release S1P in a stimulus-dependent manner, although the concentration of plasma S1P is not altered in NF-E2-deficient mice, which lack circulating platelets, or in anti-GPIba antibody-treated mice, which suffer from thrombocytopenia [19], [21], [23], [33]. The thrombin-induced S1P release from platelets isolated from SPNS2-deficient mice was also comparable to that of wild-type mice (Figure 6B). The numbers of erythrocytes and platelets in the blood of wild-type and SPNS2-deficient mice were almost equivalent (Figure 4F and 4G, and Table S1). Furthermore, *Spns2* transcripts were below the level detectable by quantitative real-time PCR. These results indicate that SPNS2 is not involved in S1P production and S1P supply from erythrocytes and platelets.

### SPNS2 is an S1P transporter of vascular ECs

**Figure 7:**
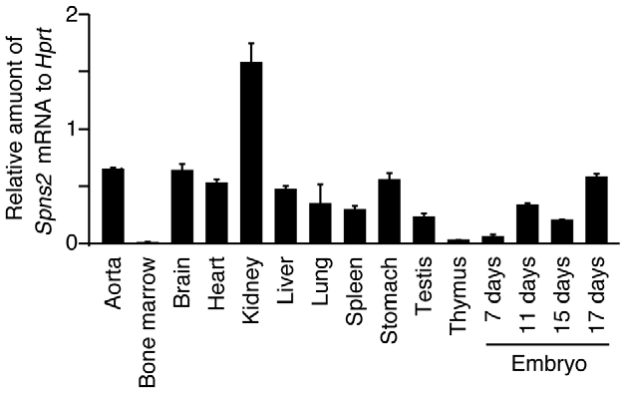
Tissue distribution of *Spns2* mRNAs. Quantitative real-time PCR was performed with first strand cDNA synthesized from mRNAs of various mouse tissues. The amount of *Spns2* mRNA in each tissue is shown relative to that of *Hprt*. The primers and probes used for PCR are given in Table S2. The graph shows the average values from four experiments, with error bars representing the standard error.

**Figure 8:**
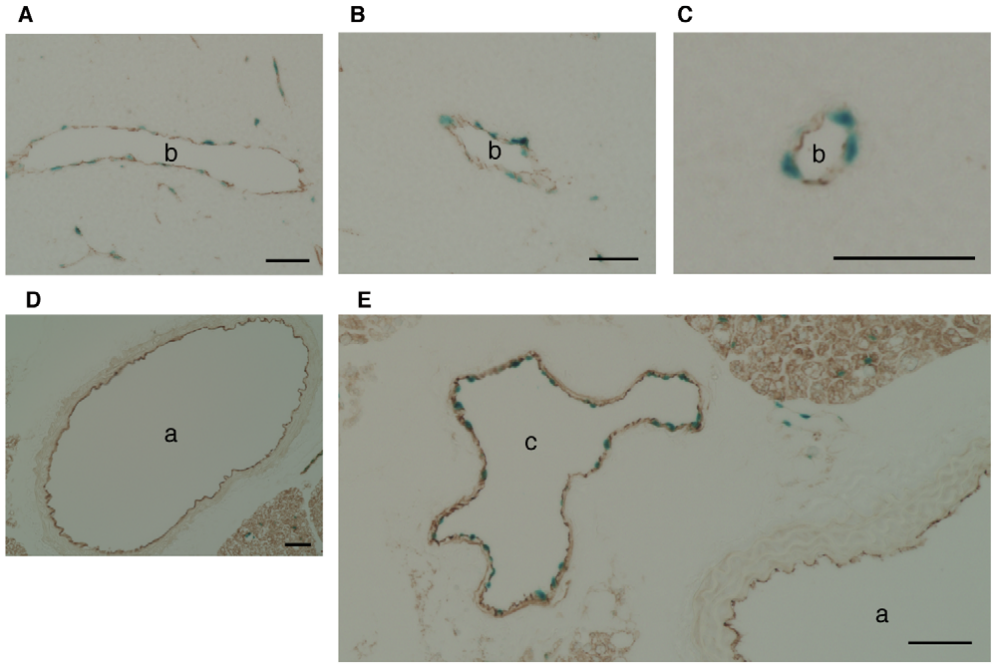
Immunohistochemical analysis of thymus. (A–C), aorta (D) and cava (E) sections from SPNS2-heterozygous mice, stained with X-gal (blue) and immunostained with CD31 antibody (brown). a, aorta; b, blood vessel; c, cava, bar, 50 µm.

**Figure 9:**
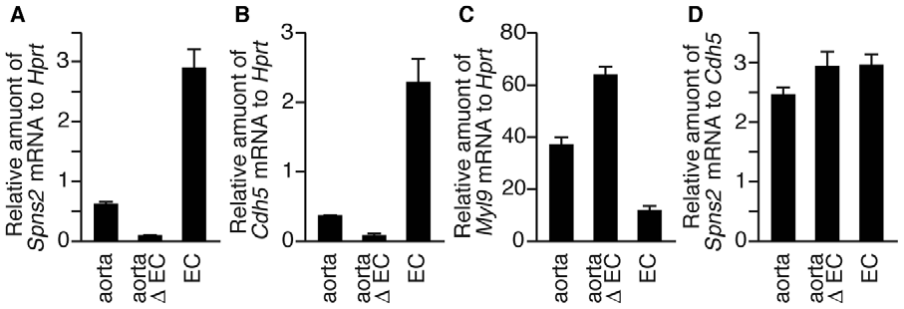
*Spns2* mRNA is expressed in aortic ECs. Quantitative real-time PCR was performed with first strand cDNA synthesized from mRNAs of the aorta. The relative amounts of *Spns2* (A and D), *Cdh5* (B) and *Myl9* (C) mRNA were measured using total RNA prepared from mouse whole aorta (aorta), aorta where ECs were removed by the collagenase-treatment (aorta Δ EC) or ECs which were recovered from that aorta (EC). *Cdh5* and *Myl9* were used as ECs and smooth muscle cell marker genes, respectively. The primers and probes used for PCR are indicated in Table S2. The graph shows the average values from four experiments, with error bars representing the standard error.

We examined whether vascular ECs utilize SPNS2 for S1P secretion because it was reported that human umbilical vein ECs (HUVECs) are able to release S1P into the culture medium [19]. In fact, *Spns2* mRNA was detected in ECs of peripheral blood vessels in the thymus and kidney (Figure S3). The transcription of *Spns2* mRNA was limited to ECs and was not detected at all in other cells, including blood cells. Although *Spns2* mRNA was detected in the aorta by quantitative real-time PCR, it was not detected by *in situ* hybridization in ECs of the aorta or the cava (Figure 7 and Figure S3K). Because the targeting vector for the SPNS2-deficient mice was designed to replace *Spns2* with the *lacZ* gene, tissues from *Spns2*-heterozygous mice were stained with X-gal to identify the SPNS2-expressing cells. CD31-positive ECs were clearly stained with X-gal in the thymus, similar to the *in situ* hybridization results. Furthermore, the signals were observed in ECs of the cava (although not detected by *in situ* hybridization) but not in the aorta (Figure 8). These results suggest that there were enough *Spns2* transcripts present in the aorta for detection with quantitative real-time PCR, but the level of transcript was below the sensitivity of *in situ* hybridization and X-gal staining. To confirm the transcription of *Spns2* mRNA in aortic ECs, quantitative real-time PCR was performed using the mRNAs from whole aorta and from ECs-depleted aorta. The amount of *Spns2* mRNA was significantly decreased in the ECs-depleted aorta (Figure 9). The relative transcription of *Spns2* mRNA was higher in the ECs prepared from aorta. When the amount of *Spns2* mRNA was normalized to that of *Cdh5*, an EC marker gene, the expression level was nearly equivalent, suggesting that the amount of *Spns2* mRNA is dependent on that of ECs (Figure 9). These results indicate that *Spns2* mRNA is transcribed in aortic ECs, although at a low level.

**Figure 10:**
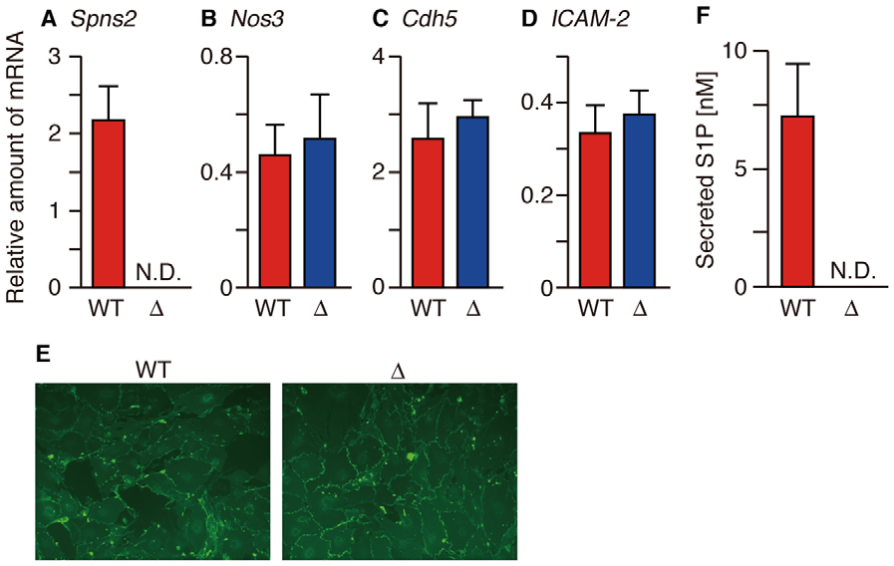
SPNS2 is an S1P transporter of vascular EC. (A–D) The relative amount of the indicated mRNAs in MAECs isolated from wild-type (WT) and SPNS2-deficient mice (Δ). The amount of each mRNA was normalized to that of *Hprt*. (E) CD31 expression by MAECs was detected by immunostaining with CD31 antibody. (F) The amount of endogenous S1P released from MAECs. The cells were incubated with 1% BSA for 4 hr at 37 °C, and the released S1P was measured by UPLC-MS/MS. The graphs show the average values from three experiments, with error bars representing the standard error. N.D., not detected.

Mouse aortic ECs (MAECs) were isolated from wild-type and SPNS2-deficient mice to measure their S1P release activity. *Spns2* mRNA was detected in MAECs isolated from wild-type but not SPNS2-deficient mice (Figure 10A). Cell morphology, the expression of the EC surface marker CD31 and the mRNA levels of other EC-specific markers (*Nos3*, *Cdh5* and *Icam-2*) were similar in both genotypes, as determined by immunostaining and quantitative real-time PCR analysis (Figure 10B–10E) [36]. MAECs prepared from wild-type mice showed S1P release activity, while SPNS2-deficient MAECs completely lost activity (Figure 10F), indicating that SPNS2 is the sole S1P transporter of MAECs.

**Figure 11:**
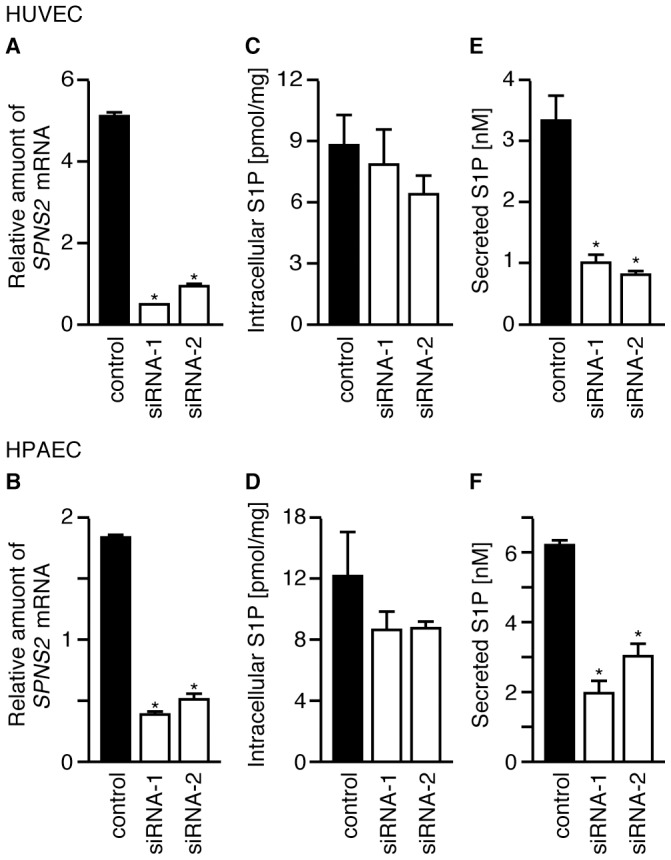
SPNS2 releases S1P from human vascular ECs. HUVECs and HPAECs were treated with two siRNAs targeting *SPNS2* mRNA or with a negative control siRNA. (A and B) Relative amount of *SPNS2* mRNA in cells treated with siRNA. Total RNA was isolated, and *SPNS2* and *GAPDH* mRNA levels were determined by quantitative real-time PCR. The amount of *SPNS2* mRNA is normalized to *GAPDH* mRNA. (C and D) Intracellular S1P. (E and F) The cells were incubated with 1% BSA for 4 hr at 37 °C, and the released S1P was measured by UPLC-MS/MS. The cells were collected, and the intracellular S1P content was measured by HPLC. C_17_-S1P was used as the internal standard. The graphs show the average values from three (C and E) or four (A, B, D and F) experiments, with error bars representing the standard error. *P<0.005 compared to ‘control’.

We also examined whether SPNS2 functions as an S1P transporter in human vascular ECs, such as HUVECs and human pulmonary artery ECs (HPAECs), derived from venous and arterial endothelia, respectively. When HUVECs or HPAECs were treated with *SPNS2*-specific siRNAs, the expression of *SPNS2* mRNA decreased to less than 20% of the control (Figure 11A and 11B). To investigate the off-target effect of these siRNAs, the expression of four ABC transporters was examined because ABCA1, ABCB1, ABCC1 and ABCG2 have been reported to play a role in S1P release from the cells [25], [27], [37], [38], [39], [40]. We confirmed that the expression of these ABC transporters was not changed between the siRNAs targeting *SPNS2* and the negative control (Figure S4). The amount of secreted S1P was significantly decreased, while the amount of intracellular S1P was not altered, in HUVECs or HPAECs treated with siRNAs targeting *SPNS2* (Figure 11C–11F). Because intracellular S1P should be rigorously controlled by various sphingolipid metabolizing enzymes such as SPHKs, SPL and SPPs, the intracellular S1P concentration should show no significant change regardless of the deletion of S1P secretion activity. These results indicate that SPNS2 plays a central role in releasing S1P from ECs in mice and humans.

## Discussion

In previous reports, we identified zebrafish Spns2 as a physiological S1P transporter and revealed that human SPNS2 also transports S1P and its analogs [28], [29]. Although there is no significant difference in enzymatic properties between zebrafish Spns2 and mammalian SPNS2, YSL is a fish-specific tissue where zebrafish Spns2 has a physiological function and supplies S1P for the regulation of myocardial precursor migration [28]. In this report, we aimed to identify the cells where mammalian SPNS2 functions as an S1P transporter.

Analysis of the cells isolated from SPNS2-deficient mice demonstrated that mammalian SPNS2 is the S1P transporter in vascular ECs but not in erythrocytes and platelets. Consistent with previous reports indicating a predominant role of erythrocytes in maintaining the plasma S1P level [22], SPNS2-deficient mice retain 60% of plasma S1P, which is likely supplied by unidentified S1P transporter(s) in erythrocytes. Among blood cells, leukocytes do not release S1P at all, but mast cells can release S1P [22], [26], [37]. The contribution of mast cells to plasma S1P levels, however, does not seem to be significant, due to the relatively low number of these cells in blood [41], [42], [43], [44]. Therefore, we believe that the decrease in plasma S1P level and circulating lymphocytes in SPNS2-deficient mice might be caused by the dysfunction of SPNS2 in vascular ECs, although the mice used in this study were not vascular endothelial cell-specific knock-out mice.

Although *Spns2* mRNA was detected in peripheral blood vessels but not in the aorta or the cava by *in situ* hybridization, transcripts derived from the *Spns2* locus were detected in ECs of the cava but not the aorta by X-gal staining (Figure 8 and Figure S3). However, quantitative real-time PCR indicated that *Spns2* mRNA was transcribed in aortic ECs (Figure 9). HUVECs derived from veins have more *Spns2* mRNA than HPAECs derived from the aorta (Figure 11A and 11B). Taken together, there might be differences in the expression level of SPNS2 among different regions of blood vessels, and SPNS2 might have a tendency to be expressed at higher levels in venous ECs.

To date, four factors concerned with S1P signaling have been reported to be essential for thymocyte egress: SPHKs for production of S1P, SPL and LPP3 for maintaining low S1P levels in the thymus and S1P1 for recognition of the signal [14], [15], [21], [45], [46], [47]. SPHKs produce S1P in intracellular spaces, and S1P1 on thymocytes recognizes S1P in extracellular spaces. Because high S1P levels cause S1P1 internalization and a defect of thymocyte egress, extracellular S1P concentration needs to be strictly regulated. The intracellular and extracellular S1P is degraded by SPL and LPP3, respectively, resulting in the optimal extracellular S1P concentration for thymocyte egress. Blood vessels are the exit for thymocytes from the thymus to the blood. Thus, vascular ECs may contribute to the S1P supply of the extracellular space of the thymus. In SPNS2-deficient mice, the S1P concentration of the extracellular environment around peripheral vascular endothelial cells in the thymus may be decreased, and mature thymocytes may not recognize S1P to exit, although the total S1P concentration of the thymus was not changed (Figure 3). We proposed that SPNS2 is a novel fifth factor involved in thymocyte egress.

In addition to lymphopenia, SPNS2-deficient mice show an EOB phenotype, suggesting that SPNS2 also functions in cells other than the vascular ECs. The EOB phenotype seems to be due to the dysfunction of the migration of eyelid cells at the embryonic stage, as shown in c-Jun- or LGR4-deficient mice [48], [49], [50]. A correlation between any S1P receptors function and eyelid migration has not been reported. S1P signaling through receptors participates in the migration of myocardial precursors, osteoclast precursors and various types of cells [5], [28], [51], [52]. Although extensive experimentation is necessary to elucidate the physiological roles of S1P, it is possible that the contribution of S1P signaling to eyelid cell migration could be demonstrated by further analysis of the SPNS2-deficient mice. Furthermore, analysis of both secretion and signal-receiving processes would be useful for elucidating the complete picture of S1P signaling.

Using SPNS2-deficient mice, we have demonstrated that SPNS2 regulates plasma S1P levels and is indispensable for thymocyte egress. S1P signaling is essential for the migration of various cell types such as lymphocytes, preosteoclasts and ECs. These results, along with our previous observations that zebrafish Spns2 mutants are defective in myocardial precursor migration [28], indicate that SPNS2 is a common regulator of the migration of cells expressing S1P receptor.

During the reviewing process of this manuscript, it was reported (Fukuhara S. et. al. *J. Clin. Invest.* **122**, 1416–1426 (2012) ) that SPNS2 expressed in endothelial cells regulates lymphocyte trafficking in mice.

## Materials and Methods

### Reagents

Antibodies against CD31 were from BD Biosciences (MEC 13.3) and Spring Bioscience (polyclonal antibody). Antibodies against CD8 (53-6.7), CD4 (RM4-5), B220 (RA3-6B2) and CD62L (MEL-14) were from BioLegend, Inc. Bovine serum albumin (fatty acid-free) (BSA) and sphingosine were from Sigma. S1P and C_17_-S1P were from Avanti.

### Mice

SPNS2-deficient mice were generated by Deltagen, Inc. (San Mateo, CA, USA) by replacing part of exon 6 and exon 7 of the mouse *Spns2* gene with a lacZ-Neomycin cassette (Figure 2). This deleted region contains the codon encoding the Arg200 that is essential for the S1P export activity of SPNS2 (Figure 1 and 2). SPNS2-deficient mice were backcrossed onto the C57BL/6 background for ten generations to create a congenic strain. Genotyping of SPNS2-deficient mice was performed by PCR against genomic DNA isolated from the tail of each mouse. PCR conditions and primers were as follows: 30 cycles of denature (96°C, 10 sec), annealing (60°C, 30 sec), and extension (68°C, 90 sec) using SPNS2-F, GGTCCTCCAGAATTCTCTGTTCTCC; Neo-F, GGGCCAGCTCATTCCTCCCACTCAT and SPNS2-R, TTGTGGCAGTTCACACTTACCTGCC. Wild-type mice used as control in this study were littermates of the SPNS2-deficient mice. Mice were housed under conventional conditions at the animal care room at ISIR, Osaka University. All experimental procedures followed the regulations of the Institutional Animal Care and Use Committee of ISIR, Osaka University (approval # 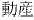 19-02-1).

### MAECs

MAECs were isolated according to the methods of Kobayashi *et al.* with some modifications [36]. Briefly, the mouse aorta was dissected out from the aortic arch to the abdominal aorta and immersed in 20% FBS-DMEM containing 100 units/ml heparin, 100 units/ml penicillin-G and 100 µg/ml streptomycin. A 24-gauge cannula was inserted into the proximal portion and the distal end was closed with a silk thread and filled with collagenase type II solution. After incubation for 45 min at 37°C, ECs were removed from the aorta by flushing with 2 ml of 20% FBS-DMEM, resuspended with 20% FBS-DMEM and cultured in a 24-well collagen type I-coated plate. To remove smooth muscle cells, after 2 hr of incubation at 37°C, the ECs were washed with warmed 20% FBS-DMEM and cultured in medium G until confluent.

### Human vascular ECs

HUVECs and HPAECs were purchased from Cell Applications, Inc. These cells were cultured following the manufacturer's instructions.

### Flow cytometry

Cell suspensions from the thymus were prepared by mincing organs in RPMI 1640 medium and then passing the cells through a nylon mesh. Erythrocytes were removed from blood samples by incubation in Lysis buffer (BD Biosciences). Isolated cells were labeled with the antibodies and analyzed using Guava easyCyte 8HT.

### Separation of single positive thymocytes

CD4 or CD8 single positive cells were purified from the thymus of mice by negative and positive selection using specific MACS microbeads conjugated to anti-mouse CD4 or CD8 antibodies (Miltenyi Biotech). Briefly, for the separation of CD4 single positive cells, thymus cell suspensions were labeled with CD8 MicroBeads and then passed over an LD column. The negatively selected cells were treated with CD4 MicroBeads and then passed over an LS column. CD4 single positive cells were enriched in the positively selected cell fraction. For the separation of CD8 single positive cells, the opposite of the selection protocol for the CD4 single positive cell selection was performed; CD4-positive cells were depleted and CD8-positive cells were collected. The separated cell populations were analyzed by flow cytometry, and the purity was more than 80%.

### Chemotaxis assays

Chemotaxis assays were performed as described previously, with slight modifications [45]. Briefly, cell suspensions from the thymus were loaded onto the upper chamber and a medium containing various concentration of S1P was added to the lower chamber of 5 µm-transwells (Corning). After 3 hr of incubation at 37°C, the cells collected from the upper and lower chambers were labeled with the anti-CD4, anti-CD8 and anti-CD62L antibodies and analyzed by flow cytometry.

### Measurement of S1P release from mouse erythrocytes

Mice were anesthetized, and blood was collected from their hearts using an acid citrate-dextrose solution (ACD) as an anticoagulant. Erythrocytes were prepared by centrifugation at 100×*g* for 15 min at room temperature and washed twice with a mixture of buffer A (20 mM HEPES-NaOH (pH 7.4), 3.3 mM NaH_2_PO_4_, 2.9 mM KCl, 1 mM MgCl_2_, 138 mM NaCl and 1 mg/ml glucose) containing 1% BSA, followed by immediate resuspension in the same buffer. S1P release from erythrocytes was measured as reported previously, with slight modifications [31]. The erythrocyte suspensions (180 µl, 1×10^7^ erythrocytes/ml) in buffer A containing 1% BSA were preincubated for 5 min at 37°C. Assay buffer containing 0.2 µM [^3^H]sphingosine (40 nCi/10 µl) in buffer A and 1% BSA was then added to each suspension (final concentration of sphingosine, 10 nM) and incubated at 37°C. After an indicated incubation period, erythrocytes and the assay buffer were separated by centrifugation at 12,000×*g* for 5 sec at 4°C. Lipids were extracted from the supernatant and erythrocytes and developed by HPTLC (Merck) in butanol/acetic acid/water (3∶1∶1 v/v). Radioactive bands were quantified with a FLA-3000G Bioimaging Analyzer (Fujifilm).

### Measurement of S1P release from platelets

Mouse blood was collected as described above, and then platelet-rich plasma (PRP) was obtained by centrifugation at 100×*g* for 15 min at room temperature. Platelets were prepared by centrifugation of PRP at 1,000×*g* for 15 min and washed with buffer A containing 1% BSA, followed by immediate resuspension in the same buffer. S1P release from platelets was measured as reported previously, with slight modifications [33], [34]. First, 190 µl of platelet suspensions (1×10^8^ platelets/ml) in buffer A containing 1% BSA were preincubated for 10 min at 37°C. Then, 10 µl of thrombin (final concentration, 5 units/ml) was added to the mixture, followed by incubation for 10 min. After incubation, the platelets and the medium were separated by centrifugation at 12,000×*g* for 5 sec at 4°C. An equal volume of methanol was added to the supernatant, samples were precipitated by centrifugation at 12,000×*g* for 5 min at 4°C, and the resulting supernatant was applied to a Cosmospin filter G and analyzed by UPLC-MS/MS.

### Measurement of S1P release from ECs

MAECs were cultured in a 24-well collagen type I-coated plate until confluent, when the number of cells was approximately 1×10^5^ cells/well. HUVECs and HPAECs were cultured in a 6-well plate for 2 days after the siRNA transfection, when the number of cells was approximately 2×10^5^ cells/well. The medium of the cultured ECs was replaced with releasing medium (endothelial cell serum-free defined medium (Cell Applications) with 1% BSA, 10 mM sodium glycerophosphate, 5 mM sodium fluoride, 1 mM semicarbazide and 20 mM HEPES-KOH (pH 7.4)). After 4 hr of incubation at 37°C, 200 µl aliquots of releasing medium were collected, and the cells were removed by centrifugation at 12,000×*g* for 5 min at 4°C. An equal volume of methanol was added to the supernatant, the samples were precipitated by centrifugation at 12,000×*g* for 5 min at 4°C and the resulting supernatant was applied to a Cosmospin filter G and analyzed by UPLC-MS/MS.

### S1P measurement by HPLC

Mouse plasma, organs and cells were assessed for *o*-phthalaldehyde (OPA) modification followed by HPLC analysis according to a modified protocol from Min *et al.* [53]. Plasma was prepared from whole blood by centrifugation at 2,000×*g* for 15 min at room temperature. C_17_-S1P (30 pmol) was added to each 100 µl aliquot of the sample as an internal standard and subjected to lipid extraction in alkaline chloroform conditions. Organs (thymus, spleen, lung and brain) were homogenized in PBS, and C_17_-S1P (30 pmol) was added to each sample. Total lipid in the organs was extracted using the Bligh-Dyer method [54] and subjected to lipid extraction in alkaline chloroform conditions. The cultured cells were washed with PBS twice, scraped and homogenized. C_17_-S1P (30 pmol) was added to each 100 µl aliquot of sample and subjected to lipid extraction in alkaline chloroform conditions. Extracted S1P was dephosphorylated with calf intestinal alkaline phosphatase (30 units) for 90 min at 37°C. The resulting sphingosine was extracted with chloroform, dried and resuspended in ethanol. The OPA modification was performed for 1 hr at room temperature. After centrifugation of the samples, a 15 µl aliquot of the sample (135 µl) was analyzed by HPLC (Hitachi) with a Cosmosil 5C 18-AR-II column (Nacalai tesque).

### S1P measurement by UPLC-MS/MS

The endogenous S1P secreted from ECs and platelets was measured by UPLC-MS/MS. The UPLC-MS/MS analysis was performed with an Acquity ultra-performance liquid chromatography liquid handling system and a Quattro Premier XE triple quadrupole mass spectrometer controlled by MassLynx (version 4.1) MS and chromatography manager software (Waters). The separation was performed on an Acquity BEH C18 analytical column (2.1 by 100 mm; particle size, 1.7 µm, Waters) using a mobile phase consisting of eluent A (water/formic acid (100∶0.1 v/v)) and eluent B (acetonitrile/tetrahydrofuran/formic acid (50∶50∶0.1 v/v)). The gradient was as follows: From t = 0 to 0.5 min A/B 50∶50, followed from t = 0.5 to 3 min by a linear gradient from A/B 50∶50 to 0∶100, then from t = 3 to 7 min A/B 0∶100, t = 7 to 7.1 min by a linear gradient from A/B 0∶100 to 50∶50 and finally from t = 7.1 to 12 min A/B 50∶50 at a flow rate of 0.300 ml/min. A Quattro premier mass spectrometer was used in the positive ion electrospray mode with a source temperature of 120°C and a desolvation temperature of 350°C. Nitrogen was used as the nebulizing, auxiliary and desolvation gas, while argon was used as the collision gas. S1P was monitored utilizing a Multiple Reaction Monitor (MRM) at *m/z* 380.31 to *m/z* 264.2. The cone voltage and collision energy were set at 24 V and 14 eV, respectively.

### Gene knock-down with siRNAs

At 24 hr before transfection, cells were plated in 6-well plates at a density that would allow them to be 50% confluent at the time of transfection. Cells were transfected with 10 nM siRNA to silence human *SPNS2* or negative control siRNA (Ambion) using Lipofectamine RNAiMAX according to the manufacturer's instructions. Experiments were performed at 48 hr after the transfection of siRNA.

### Immunostaining

MAECs were seeded on collagen type I-coated glass and confluent cells were fixed with cold methanol for 10 min. After incubation with blocking buffer (PBS containing 1% BSA and 1% FBS) for 30 min at room temperature, the antibody against CD31 (MEC 13.3) conjugated with FITC was incubated with the cells for 3 hr at room temperature. After three washes with washing buffer (PBS containing 0.1% BSA and 0.1% FBS), the cells were mounted with GEL/MOUNT and photographed with BIOREVO (Keyence).

### X-gal staining

Tissues from SPNS2-heterozygous mice were fixed overnight in 0.2% paraformaldehyde in 100 mM PIPES (pH 6.9) containing 2 mM MgCl_2_ and 5 mM EGTA, and cryoprotected in 30% sucrose containing 2 mM MgCl_2_. Subsequently, they were frozen in OCT compound and sectioned at 6–7 mm on a cryostat. The slides were rehydrated with PBS, rinsed with 100 mM phosphate buffer (pH 7.3) containing 2 mM MgCl_2_, 0.02% Nonidet P-40 and 0.01% sodium deoxycholate at 4 °C, and then stained with 100 mM phosphate buffer (pH 7.3) containing 2 mM MgCl_2_, 0.02% Nonidet P-40, 0.01% sodium deoxycholate, 5 mM potassium ferricyanide, 5 mM potassium ferrocyanide, 0.4 mM Tris-HCl (pH 7.3) and 20 µg/mL X-gal at 37 °C in the dark. The slides were then permeabilized with 0.5% Triton X-100 in PBS, treated with 3% H_2_O_2_ in methanol for elimination of intrinsic peroxidase activity, and stained with CD31 antibody (Spring Bioscience). Histofine simplestain (Nichirei) and the Liquid DAB+ Substrate chromogen System (Dako) were used for the detection of the primary antibody.

### RT-PCR

Total RNA was extracted from cells using the PureLink RNA Mini Kit (Invitrogen) according to the manufacturer's instructions. The concentration and purity of the RNA were determined spectrophotometrically by measuring the absorbance at 260 nm and 280 nm using a NanoDrop (Thermo). The mRNA was reverse transcribed using the SuperScript VILO cDNA Synthesis Kit (Invitrogen). We also used first-strand cDNA from C57BL/6J mouse tissues obtained from Genostaff Co., Ltd. for RT-PCR and quantitative real-time PCR.

### *in situ* hybridization

Paraffin embedded blocks and sections of mouse thymus, kidney and small intestine for *in situ* hybridization (ISH) were obtained from Genostaff Co., Ltd. Tissues were dissected, fixed with Tissue Fixative (Genostaff), embedded in paraffin using their proprietary procedures and sectioned at 5 µm. For ISH, tissue sections were de-waxed with xylene and rehydrated using an ethanol series and PBS. The sections were fixed with 4% para-formaldehyde in PBS for 15 min and then washed with PBS. The sections were treated with 30 µg/ml Proteinase K in PBS for 30 min at 37°C, washed with PBS, re-fixed with 4% para-formaldehyde in PBS, washed again with PBS, and placed in 0.2 N HCl for 10 min. After being washed with PBS, the sections were acetylated by incubation in 0.1 M tri-ethanolamine-HCl (pH 8.0) with 0.25% acetic anhydride for 10 min. After being washed with PBS, the sections were dehydrated through an ethanol series. Hybridization was performed with probes at 300 ng/ml in Probe Diluent-1 (Genostaff) for 16 hr at 60°C. After hybridization, the sections were washed in 5× HybriWash (Genostaff), equivalent to 5× SSC, for 20 min at 50°C and then in 50% formamide and 2× HybriWash for 20 min at 50°C, followed by RNase treatment in 50 µg/ml RNase A in 10 mM Tris-HCl (pH 8.0), 1 M NaCl and 1 mM EDTA for 30 min at 37°C. The sections were then washed twice with 2× HybriWash for 20 min at 50°C, twice with 0.2× HybriWash for 20 min at 50°C, and once with 0.1% Tween 20 in TBS (TBS-T). After treatment with 0.5% blocking reagent (Roche) in TBS-T for 30 min, the sections were incubated with anti-DIG AP conjugate diluted 1∶1000 in TBS-T for 2 hr at room temperature. The sections were washed twice with TBS-T and then incubated in 100 mM NaCl, 50 mM MgCl_2_, 0.1% Tween 20, and 100 mM Tris-HCl (pH 9.5). Staining reactions were performed with NBT/BCIP solution overnight and then washed with PBS. The sections were counterstained with Kernechtrot stain solution and mounted with CC/Mount.

### Quantitative real-time PCR

Quantitative real-time PCR was performed using the first strand cDNA as described above with the FastStart Master Mix with ROX (Roche Applied Science) using an ABI PRISM 7000 sequence detection system. Primers and probes used for quantitative real-time PCR are listed in Table S2.

### Statistical analysis

To analyze statistical significance, we used an unpaired two-tailed Student's *t*-test. We considered *P*-values<0.05 to be significant.

## Supporting Information

Figure S1 **Nucleotide and amino acid sequence of mouse SPNS2.** Nucleotide sequences of mouse *Spns2* (GenBank accession number NM_153060) are shown together with the predicted amino acid sequences. The deleted region in the SPNS2-deficient mice is indicated in the blue box. The nucleotide sequence used for the *in situ* hybridization probe is shown with a red box. The positions of the primers used for RT-PCR are indicated with arrows. The positions of the intron are indicated with arrow heads. Amino acid residues conserved between mouse and human SPNS2 are indicated with bold letters.
(TIFF)

Figure S2 **Phenotype of SPNS2-deficient mice.** (A) SPNS2-deficient mice show an eye-open at birth phenotype. Arrowheads indicate the eyes of SPNS2-deficient mice that are opened. (B) Body weight of mice at 4 weeks old. Body weight in wild-type (+/+, male n =  30, female n = 25), heterozygous (+/−, male n = 39, female n = 51) and SPNS2-deficient (−/−, male n = 28, female n = 23) mice was measured at 4 weeks. Error bars represent standard error. *P<0.005 compared to ‘WT’. (C) Survival rate of mice. Survival rate in wild-type (+/+, n = 55), heterozygous (+/−, n = 98) and SPNS2-deficient (−/−, n = 39) mice is indicated as the percent of total natal number.
(TIFF)

Figure S3 **RNA** ***in situ*** **hybridization in mouse tissue sections.** Serial sections of thymus were used for the detection of *Spns2* mRNA with an antisense *Spns2* probe (A) and ECs with a CD31 antibody (B). Serial sections of mouse thymus (C, E and D, F) were treated with antisense (C and D) or sense (E and F) *Spns2* probe. The region used for the probe is indicated in Supplemental Figure 1. Thymus sections from SPNS-deficient mice were treated with antisense *Spns2* probe (G) or *ß-actin* probe (H). Serial sections of mouse kidney were treated with antisense (I) or sense (J) *Spns2* probe. Cells in which a positive signal was detected with the antisense probe are indicated by arrowheads. Serial sections of mouse aorta were treated with antisense *Spns2* probe (K) or *ß–actin* probe (L). a, aorta, b, blood vessel, c, cava, Bar, 50 µm.
(TIFF)

Figure S4 **Relative amount of ABC transporter mRNA in human ECs after siRNA-treatment.** HUVECs (A) and HPAECs (B) were transfected with two siRNAs targeting *SPNS2* mRNA (siRNA-1 or 2) or a negative control siRNA (control). Total RNA was isolated, and mRNA levels of *ABCA1*, *ABCB1*, *ABCC1*, *ABCG2* and *GAPDH* were determined by quantitative real time PCR as described in Methods. Amount of mRNA of each ABC transporter is normalized with that of *GAPDH*. Graphs show the average values from four experiments, with error bars representing standard error.
(TIFF)

Table S1 **Results of blood analysis of wild-type and SPNS2-deficient mice.** Blood was isolated from 4–5 weeks old wild-type (WT) and SPNS2-defecient (KO) mice and used for analysis of indicated blood parameters. MCV, MCH and MCHC are Mean Corpuscular Volume, Mean Corpuscular Hemoglobin and Mean Corpuscular Hemoglobin Concentration, respectively.
(DOCX)

Table S2 **Primers and probes used for quantitative real time PCR.** Amount of the transcript for each gene (gene) was determined by the Quantitative real-time PCR using Forward primer, Reverse primer and indicated number of TaqMan probe (probe) in Roche Universal Probe Library Set.
(DOCX)
