# Supplementary figures and images for: Mouse SPNS2 Functions as a Sphingosine-1-Phosphate Transporter in Vascular Endothelial Cells
Source: PLoS One. 2012 Jun 12;7(6):e38941. doi: 10.1371/journal.pone.0038941 (PMC3379171; doi:10.1371/journal.pone.0038941)

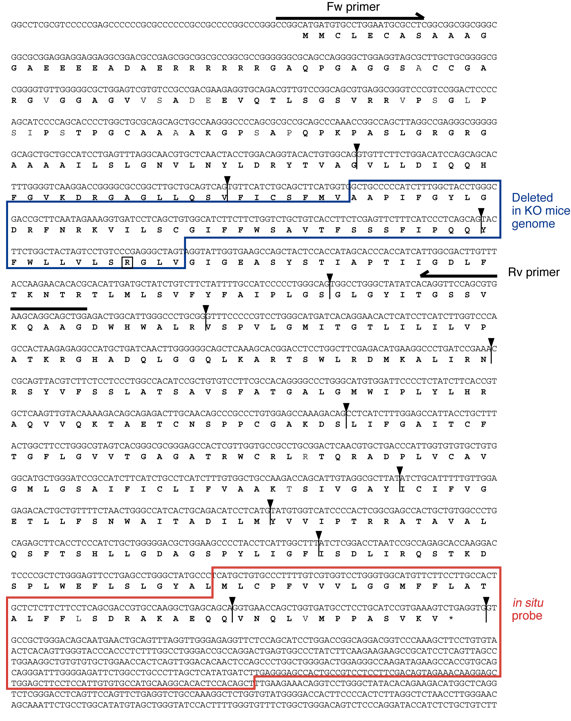

Supplement: Figure S1 — Nucleotide and amino acid sequence of mouse SPNS2. Nucleotide sequences of mouse Spns2 (GenBank accession number NM_153060) are shown together with the predicted amino acid sequences. The deleted region in the SPNS2-deficient mice is indicated in the blue box. The nucleotide sequence used for the in situ hybridization probe is shown with a red box. The positions of the primers used for RT-PCR are indicated with arrows. The positions of the intron are indicated with arrow heads. Amino acid residues conserved between mouse and human SPNS2 are indicated with bold letters. (TIFF) [file pone.0038941.s001.tiff]

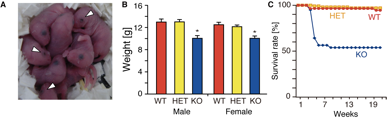

Supplement: Figure S2 — Phenotype of SPNS2-deficient mice. (A) SPNS2-deficient mice show an eye-open at birth phenotype. Arrowheads indicate the eyes of SPNS2-deficient mice that are opened. (B) Body weight of mice at 4 weeks old. Body weight in wild-type (+/+, male n = 30, female n = 25), heterozygous (+/−, male n = 39, female n = 51) and SPNS2-deficient (−/−, male n = 28, female n = 23) mice was measured at 4 weeks. Error bars represent standard error. *P<0.005 compared to ‘WT’. (C) Survival rate of mice. Survival rate in wild-type (+/+, n = 55), heterozygous (+/−, n = 98) and SPNS2-deficient (−/−, n = 39) mice is indicated as the percent of total natal number. (TIFF) [file pone.0038941.s002.tiff]

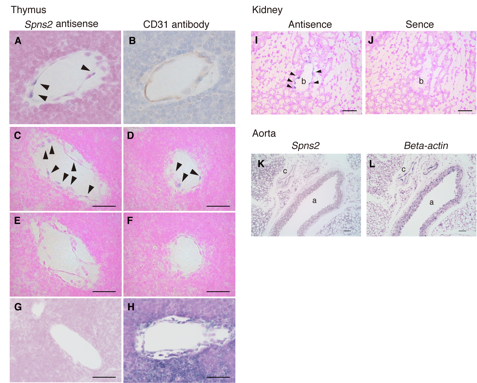

Supplement: Figure S3 — RNA in situ hybridization in mouse tissue sections. Serial sections of thymus were used for the detection of Spns2 mRNA with an antisense Spns2 probe (A) and ECs with a CD31 antibody (B). Serial sections of mouse thymus (C, E and D, F) were treated with antisense (C and D) or sense (E and F) Spns2 probe. The region used for the probe is indicated in Supplemental Figure 1. Thymus sections from SPNS-deficient mice were treated with antisense Spns2 probe (G) or ß-actin probe (H). Serial sections of mouse kidney were treated with antisense (I) or sense (J) Spns2 probe. Cells in which a positive signal was detected with the antisense probe are indicated by arrowheads. Serial sections of mouse aorta were treated with antisense Spns2 probe (K) or ß–actin probe (L). a, aorta, b, blood vessel, c, cava, Bar, 50 µm. (TIFF) [file pone.0038941.s003.tiff]

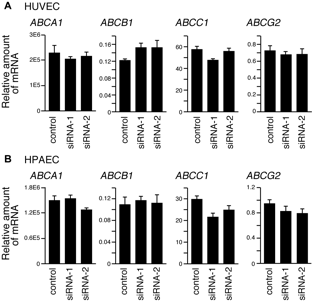

Supplement: Figure S4 — Relative amount of ABC transporter mRNA in human ECs after siRNA-treatment. HUVECs (A) and HPAECs (B) were transfected with two siRNAs targeting SPNS2 mRNA (siRNA-1 or 2) or a negative control siRNA (control). Total RNA was isolated, and mRNA levels of ABCA1, ABCB1, ABCC1, ABCG2 and GAPDH were determined by quantitative real time PCR as described in Methods. Amount of mRNA of each ABC transporter is normalized with that of GAPDH. Graphs show the average values from four experiments, with error bars representing standard error. (TIFF) [file pone.0038941.s004.tiff]
