# Supplementary material for: Mouse SPNS2 Functions as a Sphingosine-1-Phosphate Transporter in Vascular Endothelial Cells
Source: PLoS One. 2012 Jun 12;7(6):e38941. doi: 10.1371/journal.pone.0038941 (PMC3379171; doi:10.1371/journal.pone.0038941)
Supplement: Table S1 — Results of blood analysis of wild-type and SPNS2-deficient mice. Blood was isolated from 4–5 weeks old wild-type (WT) and SPNS2-defecient (KO) mice and used for analysis of indicated blood parameters. MCV, MCH and MCHC are Mean Corpuscular Volume, Mean Corpuscular Hemoglobin and Mean Corpuscular Hemoglobin Concentration, respectively. (DOCX) [file pone.0038941.s005.docx]

**Table S1. Results of blood analysis of wild-type and SPNS2-deficient mice.**

| Sex | Genotype | Leukocyte | Erythrocyte | Platelet | Hemoglobin | Hematocrit | MCV | MCH | MCHC |
| --- | --- | --- | --- | --- | --- | --- | --- | --- | --- |
|  |  | ×10^2^/μL | ×10^4^/μL | ×10^4^/μL | g/dL | % | fL | pg | g/dL |
| ♂ | WT | 28.8 ± 2.7 | 786.0 ± 21.0 | 121.4 ± 7.5 | 12.1 ± 0.4 | 41.7 ± 1.1 | 53.1 ± 0.1 | 15.4 ± 0.2 | 29.1 ± 0.3 |
|  | KO | 11.1 ± 0.7 | 752.7 ± 61.3 | 101.8 ± 9.3 | 11.7 ± 0.9 | 40.1 ± 2.1 | 53.4 ± 1.4 | 15.6 ± 0.2 | 29.1 ± 0.6 |
| ♀ | WT | 59.2 ± 15.7 | 872.3 ± 73.2 | 100.7 ± 8.5 | 13.7 ± 1.3 | 46.0 ± 4.5 | 52.6 ± 0.9 | 15.7 ± 0.2 | 29.8 ± 0.1 |
|  | KO | 15.9 ± 1.4 | 748.0 ± 36.7 | 75.2 ± 15.5 | 11.8 ± 0.9 | 40.1 ± 3.2 | 53.5 ± 2.4 | 15.7 ± 0.5 | 29.4 ± 0.6 |

(mean ± standard error)
