# Supplementary material for: Mouse SPNS2 Functions as a Sphingosine-1-Phosphate Transporter in Vascular Endothelial Cells
Source: PLoS One. 2012 Jun 12;7(6):e38941. doi: 10.1371/journal.pone.0038941 (PMC3379171; doi:10.1371/journal.pone.0038941)
Supplement: Table S2 — Primers and probes used for quantitative real time PCR. Amount of the transcript for each gene (gene) was determined by the Quantitative real-time PCR using Forward primer, Reverse primer and indicated number of TaqMan probe (probe) in Roche Universal Probe Library Set. (DOCX) [file pone.0038941.s006.docx]

**Table S2. Primers and probes used for quantitative real time PCR.**

| Gene | Forward | Reverse | Probe |
| --- | --- | --- | --- |
| human *SPNS2* | TTACTGGCTCCAGCGTGA | TGATCATGCCCAGGACAG | 27 |
| human *GAPDH* | CCCCGGTTTCTATAAATTGAGC | CTTCCCCATGGTGTCTGAG | 63 |
| mouse *Spns2* | GCACTTTGGGGTCAAGGA | CCCAGGTAGCCAAAGATGG | 83 |
| mouse *Nos3* | CCAGTGCCCTGCTTCATC | GCAGGGCAAGTTAGGATCAG | 12 |
| mouse *Cdh5* | GGCAATCAACTGTGCTCTCC | CTTCGTGGAGGAGCTGATCT | 81 |
| mouse *Icam-2* | TTGCTGGAGCCTGTCTCTTC | CTCAAAGGCCTTCTCACCAG | 26 |
| mouse *Hprt* | TCCTCCTCAGACCGCTTTT | CCTGGTTCATCATCGCTAATC | 95 |
| human *ABCA1* | GCCTGCTAGTGGTCATCCTG | CCACGCTGGGATCACTGTA | 62 |
| human *ABCB1* | ACAGAAAGCGAAGCAGTGGT | ATGGTGGTCCGACCTTTTC | 21 |
| human *ABCC1* | ACTCTCTTGGGCATCACCAC | GCATGATCCCTGAAGACTGAA | 55 |
| human *ABCG2* | TGGCTTAGACTCAAGCACAGC | TCGTCCCTGCTTAGACATCC | 56 |
| mouse *S1p1* | CGGTGTAGACCCAGAGTCCT | AGCTTTTCCTTGGCTGGAG | 66 |
| mouse *Myl9* | GATAAGGAGGACCTGCACGA | GGAACATGGTGAAGTTGATGG | 60 |
